# Spectral-Domain Optical Coherence Tomography of Preclinical Chloroquine Maculopathy in Egyptian Rheumatoid Arthritis Patients

**DOI:** 10.1155/2015/292357

**Published:** 2015-08-02

**Authors:** Riham S. H. M. Allam, Mai N. Abd-Elmohsen, Mohamed M. Khafagy, Karim A. Raafat, Sherif M. Sheta

**Affiliations:** Ophthalmology Department, Kasr Al-Ainy School of Medicine, Cairo University, Cairo 11559, Egypt

## Abstract

*Purpose*. To evaluate the role of spectral-domain optical coherence tomography (SD-OCT) in early detection of Chloroquine maculopathy in rheumatoid arthritis (RA) patients. *Methods*. 40 left eyes of 40 female rheumatoid arthritis patients who received treatment chloroquine for more than one year were recruited in the study. All patients had no symptoms or signs of Chloroquine retinopathy. They were evaluated using SD-OCT, where the Central Foveal Thickness (CFT), parafoveal thickness and perifoveal thickness, average Retinal Nerve Fiber Layer (RNFL) thickness, and Ganglion Cell Complex (GCC) measurements were measured and compared to 40 left eyes of 40 normal females. *Results*. The mean CFT was found to be thinner in the Chloroquine group (238.15 *µ*m ± 22.49) than the normal controls (248.2 *µ*m ± 19.04), which was statistically significant (p value = 0.034). The mean parafoveal thickness was lesser in the Chloroquine group than the control group in all quadrants (p value <0.05). The perifoveal thickness in both groups showed no statistically significant difference (p value >0.05) in all quadrants. No significant difference was detected between the two groups regarding RNFL, GCC, or IS/OS junction. *Conclusions*. Preclinical Chloroquine toxicity can lead to early thinning in the central fovea as well as the parafoveal regions that is detected by SD-OCT.

## 1. Introduction

The antimalarial drugs, Chloroquine (CQ) and hydroxychloroquine (HCQ), have been used in the treatment of rheumatoid arthritis with the potential risk of developing retinopathy as a serious ocular complication [1, 2]. Both antimalarial drugs cause retinopathy but differ in therapeutic and dose ranges, with hydroxychloroquine being considered a safer option. This retinal toxicity appears to be the result of an affinity to bind to melanin containing structures in the eye such as the retinal pigment epithelium [3–6]; however, the ganglion cells of the retina appeared to be the first structure to be affected in animal studies [6]. The development of retinopathy is thought to be dose related [7–10] and may be completely reversible on discontinuation of the drug at the preclinical stage [11, 12]. The patients with early retinopathy can be asymptomatic, and the fundus may remain normal for a while before any signs of maculopathy appear; hence, screening for early detection in the premaculopathy stage is recommended [12–14]. Although Chloroquine has been widely replaced by hydroxychloroquine in treatment of rheumatoid arthritis due to its wider safety margin, it is still in use in our area for socioeconomic reasons. This made the screening and early detection of retinal toxicity in our patients of paramount importance.

The screening for retinopathy in patients receiving Chloroquine involves baseline examination with visual acuity, dilated fundus examination, Amsler grid testing, and/or Humphrey 10-2 field examination test. Other testing methods include color vision testing, fundus photography, and fluorescein angiography. Recently, more sophisticated techniques have been recommended for early detection of functional and structural abnormalities [14]. Multifocal electroretinogram, fundus autofluorescence, and high resolution spectral-domain optical coherence tomography (SD-OCT) may prove to be valuable tools in early detection of Chloroquine toxicity [13]. SD-OCT is an objective, quick, and reproducible technique to examine the anatomy of the retina and optic nerve, which makes it a useful screening tool. Recent studies have shown that changes in retinal thickness and loss of outer retinal layers can be detected by SD-OCT in patients with early Chloroquine toxicity, even in areas that appeared normal on fundoscopy and perimetry [15, 16]. Detection of these changes in preclinical stages can be invaluable in screening for Chloroquine maculopathy.

In this study, we compared macular thickness using SD-OCT in patients having rheumatoid arthritis and receiving Chloroquine therapy for more than one year to a group of normal individuals to identify whether SD-OCT could be a possible tool for detecting preclinical macular toxicity in this group of patients. Average RNFL thickness and average GCC were also measured as secondary outcome measures.

## 2. Materials and Methods

### 2.1. Study Subjects

This cross-sectional observational study was performed on 80 eyes of 80 subjects recruited from the Ophthalmology and Rheumatology Outpatient Clinics, Kasr Al-Ainy School of Medicine. The study followed the principles of the Declaration of Helsinki, and ethical approval was granted from the ethical committee of Kasr Al-Ainy's Ophthalmology Department. Only the left eye from each subject was included in the study. All subjects were females between 40 and 60 years of age. Patients were divided into two groups:* Group A (Chloroquine group)* included 40 eyes of 40 patients having rheumatoid arthritis treated with Chloroquine for more than one year, with normal fundus examination. Patients having optic neuropathy (e.g., glaucoma), retinopathy, previous attacks of chorioretinitis, visual field defects involving the central 10 degrees, history of hepatic or renal impairment, or history of previous intraocular surgery were excluded from the study.* Group B (control group)*: this included 40 eyes of 40 normal women with completely normal ophthalmological examination and no history of previous intraocular surgery of any type.

### 2.2. Patient's Evaluation

After informed consent, all patients underwent full history taking with special attention to disease duration, treatment duration, daily dose, and then calculation of the cumulative dose until time of examination. Calculation of the daily and cumulative dose was done by asking the patients about how many tablets they used per day and for how many years. All our patients used the Chloroquine tablet 250 mg which is prescribed in Kasr Al-Ainy Rheumatology Clinic once per day. This means that they all shared the same daily dose but differed in the cumulative dose due to the different durations of treatment they had. 

*(i) Clinical Assessment.* Complete ophthalmologic examination was done, including pupillary reaction, best corrected visual acuity (BCVA) in LogMAR units, anterior segment assessment by slit lamp examination, intraocular pressure measurement with an applanation tonometer, and fundus examination using +20 D lens (to evaluate the periphery of the retina) and +90 D lens (biomicroscopy for evaluating the posterior pole). 

*(ii) Relevant Functional Assessment. *Color vision was tested using Ishihara pseudoisochromatic plates. Color vision was considered defective if the patient could not read correct numbers for the literates or follow the line for the illiterates in three plates or more. Wrong reading of only one letter in the plate was considered abnormal. 

*(iii) Structural Assessment.* SD-OCT scans were used to assess Central Foveal Thickness (CFT), parafoveal and perifoveal thickness in microns, and integrity of the IS/OS junction as well as the average Retinal Nerve Fiber Layer (RNFL) thickness and the Ganglion Cell Complex (GCC) evaluation, including the Global Loss Volume (GLV) and the Focal Loss Volume (FLV). In this study, spectral-domain OCT machine used was RTVue (Optovue Inc., Fremont, CA, USA) model-RT100 with algorithm version (6.11.0.12). The analyses were done according to manufacturer's software protocols. Photoreceptor inner segment/outer segment junction (IS/OS) evaluated and agreed upon by two examiners, using a horizontal High Definition (HD) line scan passing through the fovea. Other scans used were Electronic Macular Map 5 mm (EMM5), which is a 5 × 5 mm square grid centered on fixation; the grid spacing, which is 0.25 mm in the inner 3 × 3 mm area and 0.5 mm in the outer area; Retinal Nerve Fiber Layer (RNFL) analysis, which includes four circular scans with a 3.45 mm diameter centered on the disc; the Ganglion Cell Complex (GCC) analysis, which is one horizontal line with a 7 mm scan length followed by 15 vertical lines with a 6 mm scan length and a 0.5 mm interval and centered one millimeter temporal to the fovea. 3D disc is 24 radial lines with 3.4 mm scan length followed by 6 concentric rings, all centered at the optic disc. GLV measures the average amount of GCC loss over the entire GCC map, based on the fractional deviation (FD) map. FLV measures the average amount of focal loss over the entire GCC map and is based on both the FD map and the pattern deviation (PD) map.

### 2.3. Outcome Measures

*(i) Primary Outcome Measures.* CFT, parafoveal thickness, and perifoveal thicknesses in all quadrants were compared in both study groups. 

*(ii) Secondary Outcome Measures.* Secondary outcome measures as as follows: integrity of the IS/OS junction, the average RNFL thickness and the GCC measurements, correlation between the OCT parameters and the cumulative dose, and duration of treatment.

### 2.4. Statistical Analysis

The data were collected in Excel spreadsheets (Microsoft Corporation, WA, USA). All statistical analyses were done using IBM SPSS v20.0 statistical software (IBM Corporation, NY, USA). Descriptive statistics were calculated, and the data were summarized as mean ± SD for numerical data and percentages for categorical data. Associations between categorical data were analyzed by Chi-square test or Fisher's exact test. Comparison between numerical variables was done by independent samples *t*-test. Correlations between different numerical variables were done by Pearson's correlation coefficient (*r*). The results were considered statistically significant with a *p value* ≤ 0.05.

## 3. Results

All subjects entered in the study were females. The clinical data from both study groups are summarized in Table 1. The rheumatoid arthritis patients were with mean age of 49.95 ± 7.78 years and mean disease duration of 8.1 ± 7.67 years. The patients were receiving treatment with Chloroquine for mean treatment duration of 3.8 ± 2.79 years (range from 1 to 13 years) and a mean cumulative dose of 346.6 ± 225.24 grams (range from 91.25 to 1186.25 grams). All the patients were asymptomatic with normal ophthalmic examination.

### 3.1. Optical Coherence Tomography

#### 3.1.1. Central Foveal Thickness (CFT)

**Figure 1:**
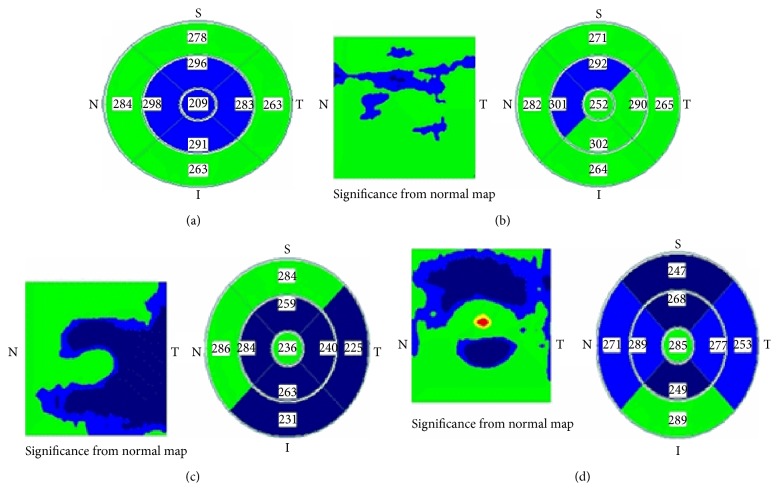
EMM5 maps from some patients in the Chloroquine group (Green is within normal; Blue is below normal). (a) EMM5 map showing thinning in the CFT as well as all parafoveal quadrants. (b) Parafoveal affection (c and d) parafoveal with perifoveal affection.

The CFT (1 mm from the foveola) in the Chloroquine group showed statistically significant thinning (Table 2, Figure 1(a)) as compared to the control group with a *p* value of 0.034.

#### 3.1.2. Parafoveal Thickness

The parafovea (3 mm from the foveola) in the Chloroquine group showed thinning (Figures 1(b) and 1(c)) as compared to the control group with high statistical significance in all quadrants (Table 2).

#### 3.1.3. The Perifoveal Area Thickness

The perifoveal area showed thinning in patients treated with Chloroquine for RA which was of no statistical significance as compared to the control group (Table 2, Figures 1(c) and 1(d)).

No significant difference was found between the two groups included in the study regarding RNFL thickness, GCC thickness, FLV, or GLV (Table 2). In addition, no statistically significant correlation was detected between either the cumulative dose of Chloroquine or the duration of the treatment with any of the parameters measured by OCT in the Chloroquine study group (Table 3).

#### 3.1.4. Photoreceptor Inner Segment/Outer Segment (IS/OS) Junction

**Figure 2:**
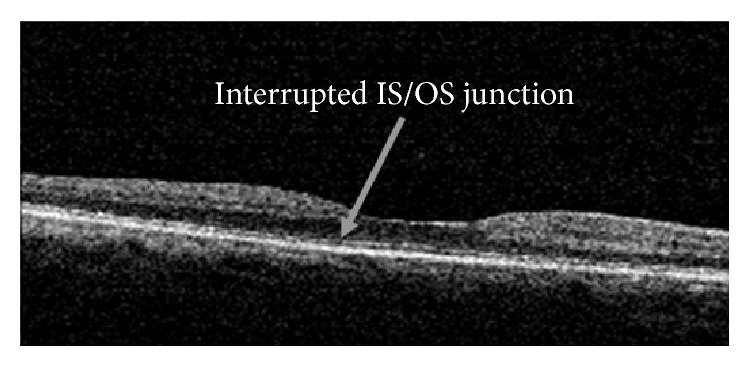
IS/OS interrupted in a patient from the Chloroquine group.

IS/OS junction showed an interruption in 2 patients from the control group and 4 patients from the Chloroquine group (Figure 2). This was statistically not significant (*p* value 0.675, Fisher's exact test).

## 4. Discussion

In this study, we have found that clinically asymptomatic patients, receiving Chloroquine treatment for rheumatoid arthritis, had decreased Central Foveal Thickness (CFT) and parafoveal thickness in all quadrants as compared to the normal controls. This change was not found to correlate with either the cumulative dose or the duration of treatment. The inner retinal layers appeared to be not affected by the Chloroquine toxicity with no difference between the RNFL and GCC SD-OCT parameters in both study groups.

Previous studies on symptomatic patients receiving Chloroquine or hydroxychloroquine therapy reported retinal thinning and loss of outer retinal layers with early retinal toxicity [15, 17]. The loss in full retinal thickness has been found to precede the changes in individual layers of the retina, such as the photoreceptor inner segment/outer segment junction loss [17]. Many studies reported the retinal thinning affecting mainly the parafoveal regions on SD-OCT [17–19]. This finding is thought to be due to loss in pericentral outer nuclear layer, photoreceptors layer, and retinal pigment epithelium (RPE) abnormalities [20, 21]. This loss in outer nuclear layer was found to correlate with functional loss detected by multifocal electroretinogram [20]. However, these studies were done on clinically symptomatic patients. The current study confirms that these findings could be detected in asymptomatic patients in the preclinical stage with normal ophthalmic examination.

The CFT was found to differ significantly between both groups in the study. To the best of our knowledge, this finding was not reported before in preclinical Chloroquine or hydroxychloroquine maculopathy, although multifocal electroretinogram (mf-ERG) abnormalities were detected at the fovea in the asymptomatic stage [14]. This may indicate early affection of the cone photoreceptors at the fovea in Chloroquine toxicity, which may be due to its narrow safety margin. We found that six patients developed central foveal thinning only (in a circle 1 mm from the fovea), four patients developed parafoveal thinning sparing the central area, and five patients showed combined central foveal and parafoveal affection. The four parafoveal quadrants in our study group were almost similarly affected with the superior quadrant most frequently affected. The perifoveal quadrants were not widely affected, but the temporal quadrant showed maximal affection. Significant loss of perifoveal retinal thickness has been reported in preclinical hydroxychloroquine maculopathy in some studies [18, 22], but this was not found in our group of patients. The extent of damage in the macular area is thought to be related to ganglion cell distribution, as suggested by primate studies [14]. Furthermore, the binding of Chloroquine to melanin pigment in the RPE and presence of an avascular zone at the center of the fovea has been suggested as a possible explanation for the distribution of damage [22]. Fundus autofluorescence is sensitive to areas of RPE loss even in very early stages, showing alternating rings of central mottled hypoautofluorescence and pericentral mottled hyperautofluorescence [14]. This needs further future research with a larger number of patients to determine where toxicity develops earlier and standardize the technique for screening.

Recent reports suggested that selective RNFL loss and innerretinal loss could be detected in preclinical patients [19, 22, 23]. However, the RNFL and GCC thickness were not found to be affected in preclinical patients in our study. This agrees with what was reported by Pasadhika and Fishman [23] that although loss of inner retinal layers (RNFL and GCC) could be detectable on SD-OCT early in hydroxychloroquine toxicity, this finding was not found in their preclinical patients. They suggested that the presence of a considerable number of nerve fibers that originate outside the macula in the RNFL contributes to the overall RNFL thickness and could explain the absence of significant RNFL loss in the premaculopathy stage when compared to healthy individuals [23]. Thus, the loss in the innerretinal layers by SD-OCT could be helpful for follow-up over time of chronic patients to detect early macular changes compared to baseline measurements [23].

The IS/OS junction loss is considered a confirmed sign of damage in early retinopathy [16, 24], and Stepien et al. [16] described a “moth-eaten” photoreceptor IS/OS junction due to hydroxychloroquine toxicity in the absence of clinical signs or field defects. They explained this finding by preferential loss of cone photoreceptors [16]. However, the study was done on four eyes only with no comparison with normal controls. The IS/OS junction loss was not found to differ significantly between our group of patients and controls. This could be attributed to the early preclinical stage of our group of patients.

The cumulative dose of Chloroquine was previously considered to be of significance as a risk factor for development of maculopathy. This was contradicted by recent studies that suggested that the daily intake adjusted to lean (ideal) body weight (actual body weight—body fat) is a more important risk factor [14, 25, 26]. The lean body weight depends on the actual body weight in kilograms factored for height in inches and its calculation differs between males and females [26]. Exclusion of the body fat while calculating the dose for Chloroquine may be crucial in obese patients to avoid overdosage, since little of the antimalarial drug is distributed into the body fat, bone, and brain [25]. Patients receiving a daily dose more than 3 mg/kg of Chloroquine or more than 6.5 mg/kg of hydroxychloroquine are considered at high risk to develop retinopathy [25–27], although some reports suggest that some patients may be affected at lower daily doses of the drugs [14, 27]. All of our patients were taking a fixed daily dose of Chloroquine 250 mg per day and there was no correlation between the cumulative dose and the foveal and parafoveal thinning seen in our patients.

In the study, we avoided patients with systemic diseases other than rheumatoid arthritis. This was done to limit the effect of other autoimmune diseases on the macula, such as systemic lupus erythematosus. Additionally, none of our patients had liver or kidney impairment. These conditions are known to increase the risk for development of maculopathy through the retention of the drug in the body, since Chloroquine depends mainly in its metabolism and excretion on the liver and kidney [26–28]. Antimalarials are known to be metabolized and stored in the lysosomes of the hepatocytes and kidney cells with potential toxic effect on both organs due to oxidative stress, with Chloroquine being about three times as toxic as hydroxychloroquine [29]. All patients were treated by the same drug, Chloroquine, with a fixed daily dose (250 mg) with no history of significant renal or hepatic impairment. This helped to limit the effect of liver and kidney condition on the drug dosage and therapeutic effect. These considerations add to the reliability of the results.

The limitations of our study may be that a greater number of patients could have been included, in combination with other functional testing modalities such as the multifocal electroretinogram or autofluorescence testing, and assessment of doses according to lean body weight could have been considered. The mean duration of the treatment in our patients was about 3.8 years with a standard deviation of 2.7 years. This is a relatively short duration for the development of retinopathy, since the high risk for progression to maculopathy is considered for more than five years of continuous use of the drug [27, 28, 30, 31]. However, presence of detectable retinal thinning in SD-OCT with such short duration of treatment highlights the value of early objective screening for Chloroquine maculopathy.

Screening of Chloroquine/hydroxychloroquine retinopathy is challenging especially in the very early stages. The preretinopathy changes in the macula and in functional testing such as the central field testing and color vision changes may be difficult to quantify and differentiate clinically from other age-related changes. Color vision testing using the Ishara pseudoisochromatic plates is not sensitive enough to detect subtle color vision affection and more sensitive and detailed tests, such as Farnsworth-Munsell 100-hue test, may be required to detect early changes [25]. The recent recommendations suggest a combination of clinical examination (visual acuity and dilated fundus examination), automated central perimetry (10-2), and at least one of the more objective testing methods: mf-ERG, SD-OCT, and fundus autofluorescence [24, 30, 31].

In summary, patients receiving Chloroquine treatment for rheumatoid arthritis exhibit retinal thinning in the foveal and parafoveal regions when examined by SD-OCT. This could be an early sign of Chloroquine toxicity irrespective of the cumulative dose or treatment duration and before clinical symptoms or signs appear. Given the toxic nature of antimalarial drugs and the more toxic nature of Chloroquine in particular, SD-OCT could be a helpful screening tool for detection of preclinical macular toxicity in integration with other functional modalities early in the course of Chloroquine treatment.

## Figures and Tables

**Table 1:** Demographic and examination data of both groups.

		Group A	Group B	*p* value
		(Chloroquine group)	(Control group)	
Age in years	Mean ± SD	49.95 ± 7.78	51 ± 7.38	0.49^*∗*^
BCVA (LogMAR)	Mean ± SD	0.3688 ± 0.307	0.4825 ± 0.343	0.122^*∗*^
Color vision (normal/defective)	Normal	(34/6)	(36/4)	0.499^*∗∗*^
Disease duration in years	Mean ± SD	8.1 ± 7.67	N/A	—
Chloroquine treatment Duration in years	Mean ± SD	3.8 ± 2.79	N/A	—
Chloroquine cumulative dose (grams)	Mean ± SD	346.6 ± 225.24	N/A	—

^*∗*^Independent samples student's *t*-test.

^*∗∗*^Chi square test.

**Table 2:** Comparison between the different OCT parameters in both groups.

OCT (*μ*m) Mean ± SD	Chloroquine group	Control group	*p* value^*∗*^
CFT	238.15 ± 22.5	248.2 ± 19.05	0.034
Parafoveal thickness			
Superior	310.7 ± 21.64	322.35 ± 12.02	0.004
Inferior	309.05 ± 18.75	319.025 ± 11.3	0.005
Nasal	311.45 ± 14.9	320.05 ± 11.39	0.005
Temporal	299.025 ± 21.67	320.05 ± 11.39	0.013
Perifoveal thickness			
Superior	287.675 ± 19.9	286.7 ± 11.85	0.791
Inferior	279.1 ± 17.75	282.75 ± 10.34	0.264
Nasal	287.38 ± 15.82	302 ± 11.4	0.107
Temporal	278.15 ± 20.6	282.6 ± 12.144	0.243
RNFL thickness	111.4458 ± 14.6969	107.6425 ± 11.5335	0.202
GCC thickness	95.61 ± 6.385	96.1217 ± 6.1347	0.716
FLV	0.86 ± 1.04	0.60 ± 0.53	0.167
GLV	4.28 ± 3.87	3.56 ± 3.05	0.358

^*∗*^Independent samples *t*-test.

**Table 3:** Pearson's correlation coefficient (*r*) between the cumulative dose and treatment duration and the OCT parameters in the study group.

OCT	Cumulative dose in grams	Treatment duration in years
CFT	−0.013 (*p* value = 0.936)	−0.017 (*p* value = 0.918)
Parafoveal thickness		
Superior	−0.134 (*p* value = 0.409)	−0.135 (*p* value = 0.405)
Inferior	−0.048 (*p* value = 0.770)	−0.048 (*p* value = 0.768)
Nasal	0.075 (*p* value = 0.644)	0.074 (*p* value = 0.649)
Temp.	−0.188 (*p* value = 0.247)	−0.189 (*p* value = 0.244)
Perifoveal thickness		
Superior	−0.065 (*p* value = 0.689)	−0.068 (*p* value = 0.678)
Inferior	−0.259 (*p* value = 0.107)	−0.260 (*p* value = 0.105)
Nasal	0.086 (*p* value = 0.598)	0.084 (*p* value = 0.605)
Temp.	−0.253 (*p* value = 0.115)	−0.256 (*p* value = 0.111)
RNFL	−0.119 (*p* value = 0.464)	−0.120 (*p* value = 0.462)
GCC thickness	−0.003 (*p* value = 0.984)	−0.002 (*p* value = 0.989)
FLV	0.056 (*p* value = 0.730)	−0.055 (*p* value = 0.736)
GLV	0.114 (*p* value = 0.483)	−0.114 (*p* value = 0.482)
